# Supplementary material for: Topological data analysis reveals a core gene expression backbone that defines form and function across flowering plants
Source: PLoS Biol. 2023 Dec 5;21(12):e3002397. doi: 10.1371/journal.pbio.3002397 (PMC10723737; doi:10.1371/journal.pbio.3002397)
Supplement: S1 Text — Fig A. Histogram of 3-way factors of the RNAseq samples before and after downsampling. The distribution of 3-way factors for family, tissue, and stress is plotted. The 16 families, 8 tissue types, and 10 stresses equate to 1,280 unique 3-way combinations, but we only observed 195 unique combinations in our dataset. The distribution of samples from the entire dataset is shown on the left, and the distribution of samples when downsampling the 30 most common 3-way combinations is shown on the right. Raw expression data underlying the graphs in this figure can be found in S7 Dataset, and code can be found in https://zenodo.org/records/8428609 [65]. Fig B. Factor-wise frequency plots of RNAseq samples before and after subsampling. The number of samples in each family, tissue type, or stress is plotted before (top) and after (bottom) subsampling. Raw expression data underlying the graphs in this figure can be found in S7 Dataset, and code can be found in https://zenodo.org/records/8428609 [65]. Fig C. Topology of Mapper graphs generated from the subsampled data. Samples from each node in the Mapper graph are colored by plant family (A), stress (B), or tissue type (C), using the subsampled data. The overall topology and sample distribution are similar to the Mapper graphs constructed with the full, unbalanced dataset, suggesting that sample distribution is not a major factor in our analyses. Fig D. Linear regression analysis of association of surrogate variables to one batch variable (BioProject), our biological variables of interest (stress, tissue, and family), and their pairwise interactions. All surrogate variables were regressed on either each variable or interaction individually to calculate adjusted R2 values. Table A. Enrichment of GreenCut2 genes in orthogroup-mapped Arabidopsis thaliana genes and stress-/tissue-correlated orthogroup-mapped genes. The proportion of GreenCut2 genes in the all the orthogroups used in this study was compared against the proportion of [file pbio.3002397.s001.docx]

**Supporting Information 1**

**Text A**

**Confounder discussion from the Surrogate Variable Analysis**

We used Surrogate Variable Analysis (SVA) [[1]](https://paperpile.com/c/A1VBac/1adwX) to explore the effects of confounding technical variables on the publicly available SRA data assembled for this study. Briefly, we identified three primary variables of interest (tissue, stress, and family), which were fixed in the model used to estimate “surrogate variables” to minimize the amount of variability attributable to these primary variables captured by the estimated surrogate variables (see Supplementary Methods for Surrogate Variable Analysis). These surrogate variables represent unaccounted for technical variables impacting the dataset. Due to the breadth of families, stresses, and tissues analyzed, we do not have a full factorial design (i.e., there are combinations of family, stress, and tissue factor values for which there are no expression datasets). Because of this, SVA would remove variability due to our primary variables and their interactions. To get a sense of what kind of impact the surrogate variables might have on the dataset when removed, we estimated the correlation between the first order interactions between our primary variables and the surrogate variables identified by SVA. We identified 24 surrogate variables which individually captured between 53% and 98% of variation between BioProjects (Fig D in S1 Text). We also estimated the interaction terms between the tissue, family, and stress factor combinations that were present in the dataset and estimated how much of their variation was getting captured by the surrogate variables. Individual surrogate variables captured up to 14% of variation between stress conditions, up to 66% of variation between tissue conditions, and up to 63% of variation between families. For the interaction terms between primary variables, individual surrogate variables captured up to 83% of the variation between tissue and family combinations, up to 65% of the variation between stress and family combinations, and up to 71% of the variation between tissue and stress combinations. This suggests that even though stress, tissue, and family are treated as protected primary variables, there are underlying latent variables related to our primary variables and their interactions that may be important sources of biological variation being captured by the surrogate variables. Although individual surrogate variables could be selectively accounted for in downstream analyses in such a way that minimizes the removal of biological signal, this would be a highly subjective process. Moreover, due to our inability to precisely calculate the true correlation between our surrogate variables and interaction terms due to the fact that many factor combinations are missing, this would be statistically dubious as well.

Because the surrogate variables show substantial linear correlation with our primary variables and their interaction terms, the application of SVA would require eliminating substantial amounts of biological signal. Since the goal of our study is to identify heterogeneous patterns due to stress, tissue, and family within a high-dimensional gene expression dataset, SVA may not be appropriate for us to use. Alternatively, one could potentially minimize the loss of this signal by cherry-picking individual surrogate variables to include in downstream analysis, which would naturally introduce human bias. A third option would be to use an algorithm like ComBat-seq[[2]](https://paperpile.com/c/A1VBac/P25qW) that relies on explicitly defined batches, which is problematic for the present study since the closest metadata for batch available for the studies gathered on SRA is the BioProject ID’s, but these are, at best, a proxy for batches of samples and are not sufficient to assess the technical variability or noise in the data. More broadly, as discussed in[[3]](https://paperpile.com/c/A1VBac/7qDKQ), such genomic data “cleaning” methods, by their very nature, delimit the observable features of the resulting datasets to those prespecified by the investigator. In our view, this limits their utility for broad exploratory analyses of the kind described in this study. For all the above reasons, we opted to not use SVA, ComBat, or related techniques.

**Supplemental Figures:**


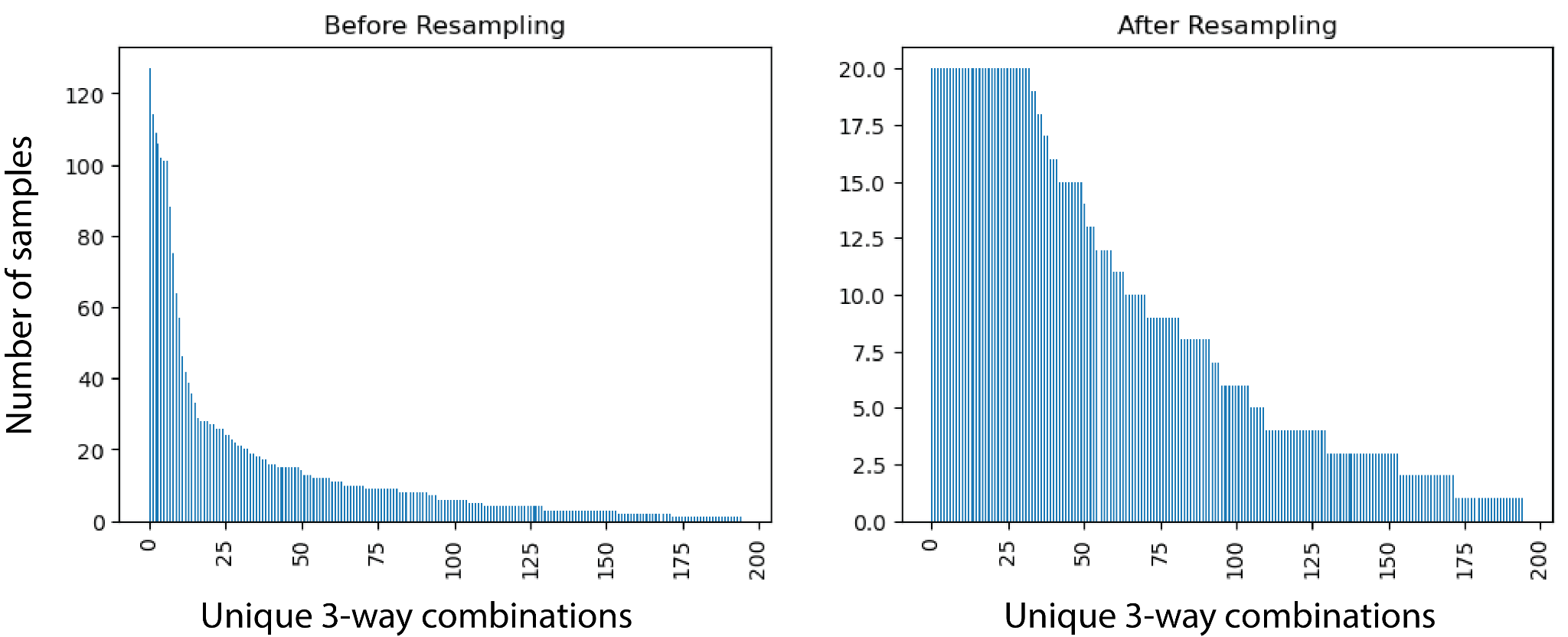


**Fig A. Histogram of 3-way factors of the RNA seq samples before and after downsampling.** The distribution of 3-way factors for family, tissue, and stress are plotted. The 16 families, 8 tissue types and 10 stresses equate to 1280 unique 3-way combinations, but we only observed 195 unique combinations in our dataset. The distribution of samples from the entire dataset is shown on the left and the distribution of samples when downsampling the 30 most common 3-way combinations is shown on the right.


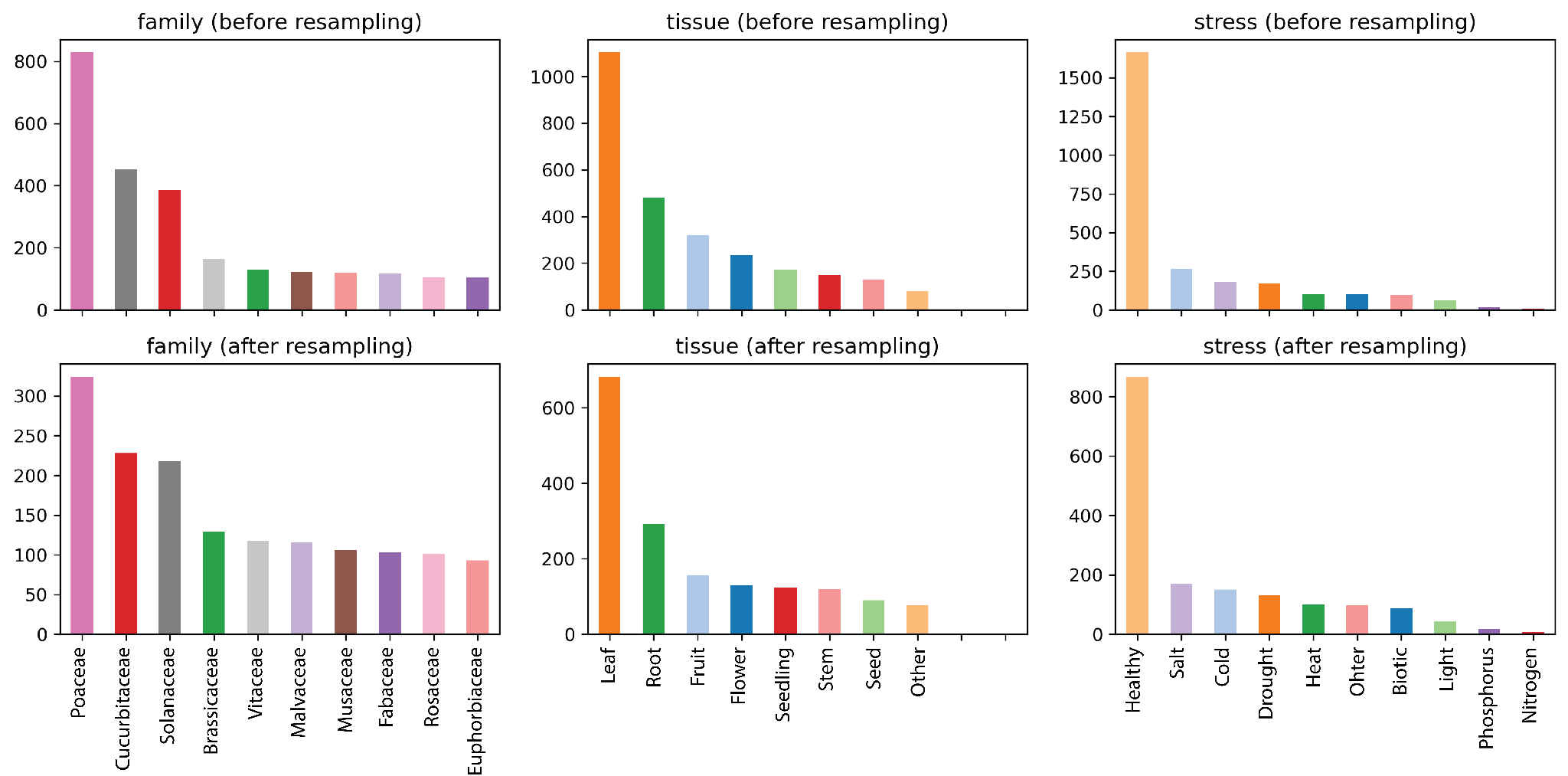


**Fig B. Factor-wise frequency plots of RNAseq samples before and after subsampling.** The number of samples in each family, tissue type, or stress are plotted before (top) and after (bottom) subsampling.
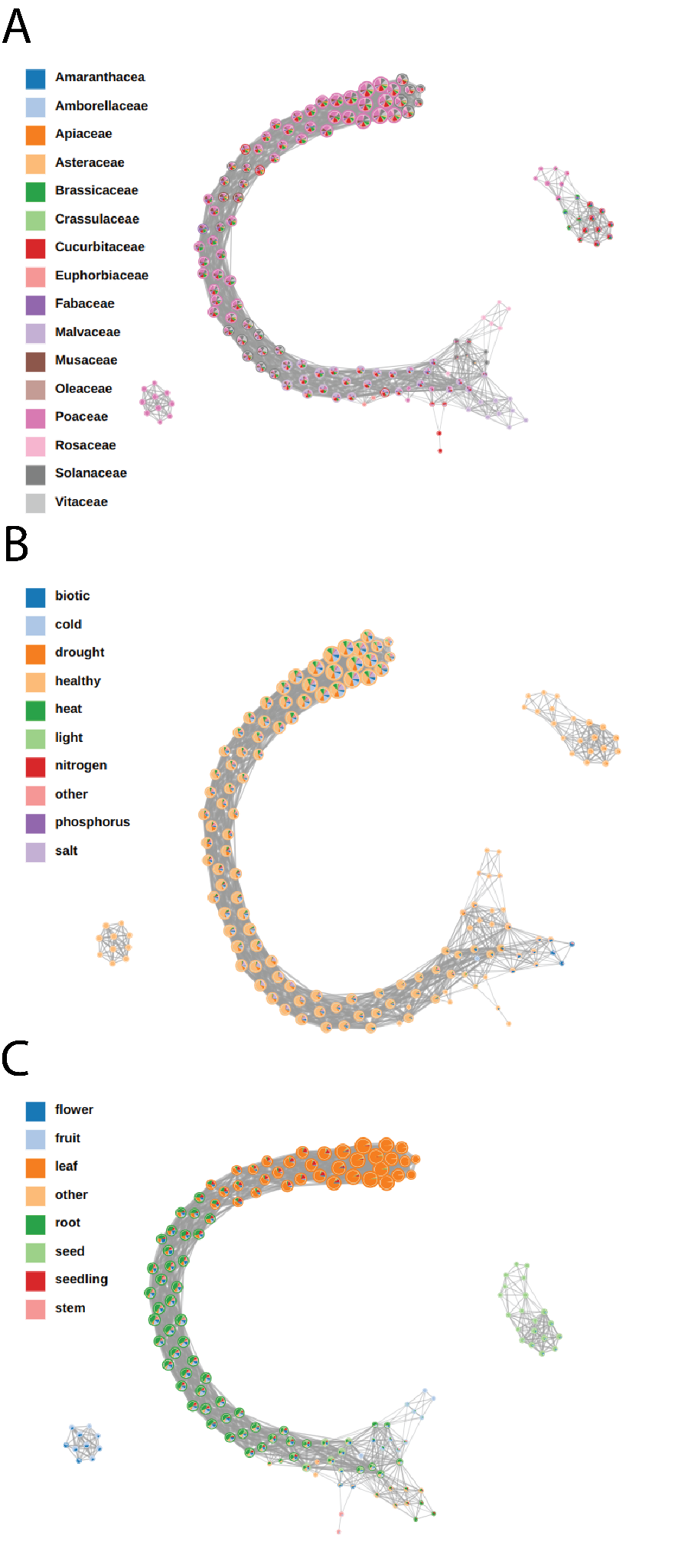


**Fig C. Topology of Mapper graphs generated from the subsampled data.** Samples from each node in the mapper graph are colored by plant family (A), stress (B), or tissue type (C), using the subsampled data. The overall topology and sample distribution are similar to the Mapper graphs constructed with the full, unbalanced dataset, suggesting sample distribution is not a major factor in our analyses.

**
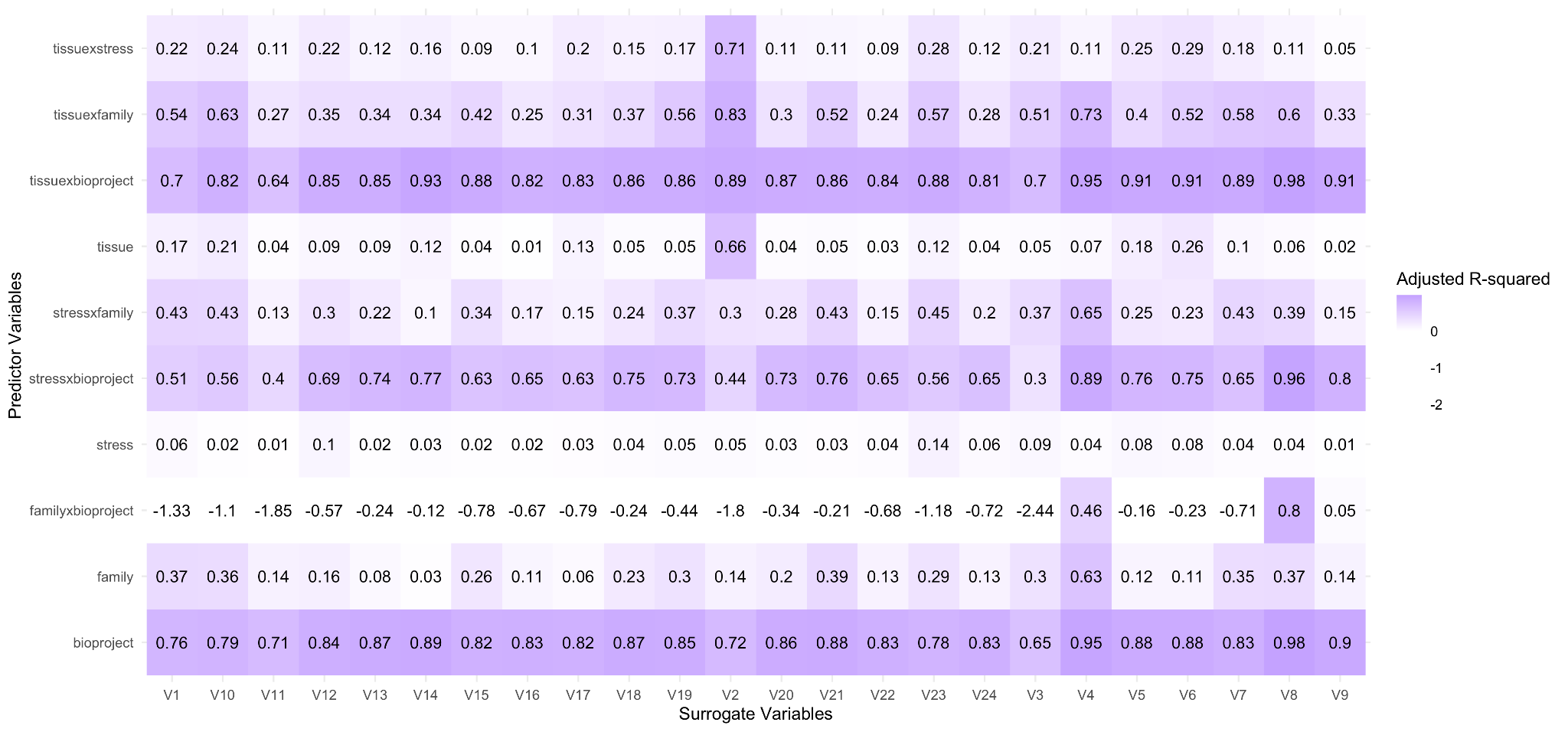
**

**Fig D.** Linear regression analysis of association of surrogate variables to one batch variable (bioproject), our biological variables of interest (stress, tissue, family), and their pairwise interactions. All surrogate variables were regressed on either each variable or interaction individually to calculate adjusted R^2^ values.

**Supplemental Tables**

**Table A.** Enrichment of GreenCut2 genes in orthogroup-mapped *Arabidopsis thaliana* genes and stress-/tissue- correlated orthogroup-mapped genes. The proportion of GreenCut2 genes in the all the orthogroups used in this study was compared against the proportion of GreenCut2 genes in a list of all *A. thaliana* genes using a one-sided binomial test. The proportion of tissue-lens and stress-lens correlated orthogroup-mapped genes in GreenCut2 was compared against the proportion of GreenCut2 genes in the entire set of orthogroup-mapped genes using one-sided binomial tests. Tissue-correlated genes were hypothesized to be more likely to be in GreenCut2 than a random selection of orthogroup-mapped genes, and the stress-correlated genes were hypothesized to be less likely.

| **Dataset** | **# of Genes in Dataset** | **# of Genes in GreenCut2** | **% GreenCut2** | **p-value** |
| --- | --- | --- | --- | --- |
| **All Arabidopsis Genes** | **27662** | **677** | **2.45** |  |
| **All Orthogroup-Mapped Genes** | **6328** | **421** | **6.65** | **2.76 * 10^-96^** |
| **All Tissue-lens Correlated Genes** | **318** | **85** | **26.7** | **9.18 * 10^-29^** |
| **Stress-lens Correlated Genes** | **318** | **7** | **2.20** | **0.000252** |

**Dataset S1 (separate file).** GO Term enrichment results on genes negatively correlated with the tissue-lens.

**Dataset S2 (separate file).** GO Term enrichment results on genes positively correlated with the tissue-lens

**Dataset S3 (separate file).** GO Term enrichment results on genes positively correlated with the stress-lens

**Dataset S4 (separate file).** GO Term enrichment results on genes positively correlated with the stress-lens

**Dataset S5 (separate file).** Overlap between orthogroup-mapped genes and tissue- and stress-lens correlated genes with the GreenCut2 resource (Karpowicz

**Dataset S6 (separate file).** Metadata of the raw data used in this experiment.

**Dataset S7 (separate file).** Expression matrix of TPMs for the normalized orthogroups

**References**

1. [Leek JT, Johnson WE, Parker HS, Jaffe AE, Storey JD. The sva package for removing batch effects and other unwanted variation in high-throughput experiments. Bioinformatics. 2012;28: 882–883.](http://paperpile.com/b/A1VBac/1adwX)

2. [Zhang Y, Parmigiani G, Johnson WE. ComBat-seq: batch effect adjustment for RNA-seq count data. NAR Genom Bioinform. 2020;2: lqaa078.](http://paperpile.com/b/A1VBac/P25qW)

3. [Jaffe AE, Hyde T, Kleinman J, Weinbergern DR, Chenoweth JG, McKay RD, et al. Practical impacts of genomic data “cleaning” on biological discovery using surrogate variable analysis. BMC Bioinformatics. 2015;16: 372.](http://paperpile.com/b/A1VBac/7qDKQ)
