# Supplementary material for: Topological data analysis reveals a core gene expression backbone that defines form and function across flowering plants
Source: PLoS Biol. 2023 Dec 5;21(12):e3002397. doi: 10.1371/journal.pbio.3002397 (PMC10723737; doi:10.1371/journal.pbio.3002397)
Supplement: Attachment — Submitted filename: Respone_to_Reviewers.docx [file pbio.3002397.s009.docx]

We thank the reviewers for their detailed comments and feedback on our manuscript. We have tried to address each of the reviewer’s comments and feel the revised manuscript is greatly improved in both its readability and technical robustness. Below are our specific responses to each comment in blue.

Reviewers' comments

Rev. 1:

In the manuscript entitled, "The topological shape of gene expression across the evolution of flowering plants", Palande et al. present an innovative new analysis method for identifying trends in large collections of data. They take transcriptomes of different tissues and under different stresses from species across the phylogeny of flowering plants and analyze them all together with topological data analysis. They represent the shape of gene expression across a tissue lens and find a continuum from leaves to seeds with some branches for specific tissues in various plant families. The genes underlying the difference in tissues generally relate to photosynthesis in leaves versus core metabolic processes such as ubiquitination and Golgi vesicle-mediated transport in non-leaf tissues. Their work on stress is less conclusive, as healthy and stressed samples are often mixed at nodes. However, they did identify a general trend from healthy to stressed. They also found a negative

correlation between stressed tissues and growth/cell division, which is a well-known trade off plants make, suggesting fundamental biology can be accessed by these analysis methods. To my knowledge this is the first use to analyze expression patterns in plants, and there are only a handful of uses in all of biology. Overall, the method is novel and adds a key new tool to analyze complex high dimensional data, particularly across species.

Major concerns

1. We ask the authors to consider and further discuss possible limitations of their method in more detail. Specifically, how does the proportion of samples in the input data affect the weight of output terms? For example, the dataset analyzed has high numbers of samples with drought, heat, and salt stress, which may explain why water deprivation comes up as a major output term for stress response overall. Something else might come up if stress samples were more balanced. Likewise, because Poaceae are a large fraction of the sample species input, they might play a big part in shaping the stress outputs. It would be worth re-running the analysis without Poaceae to see what effect that has on the output.

This is an excellent suggestion. Assembling balanced datasets is currently a major challenge when using public data, as most gene expression experiments are done in a few model or crop species with relatively narrow sampling across tissues or stresses. Balanced datasets simply do not exist for some of the factors we are exploring. To test if the unbalanced nature of our dataset is causing the patterns we observed, we downsampled the most frequent factor combinations and surveyed the effect it had on the Mapper graph topology. Our study has three factors: Family, Tissue, and Stress with 16 families, 8 tissue types and 10 stresses. In total, 1280 unique 3-way combinations are possible (family+tissue+stress), but in our dataset, only 195 unique combinations are present and they have the following distribution.

**Supplemental Figure 1. Histogram of 3-way factors of the RNA seq samples before and after downsampling.** The distribution of 3-way factors for family, tissue, and stress are plotted. The 16 families, 8 tissue types and 10 stresses equate to 1280 unique 3-way combinations, but we only observed 195 unique combinations in our dataset. The distribution of samples from the entire dataset is shown on the left and the distribution of samples when downsampling the 30 most common 3-way combinations is shown on the right.

Based on this distribution, we chose a cutoff of 30 and downsampled the 30 most common factor combinations. This significantly reduced the sampling bias for family, tissue, and stress, but it did not eliminate them, as shown below.

**Supplemental Figure 2. Factor-wise frequency plots of RNAseq samples before and after subsampling.** The number of samples in each family, tissue type, or stress are plotted before (top) and after (bottom) subsampling.

We then reran the Mapper algorithm using this downsampled dataset. The topology is quite similar, suggesting that biases in sample representation are not the major factor underlying the patterns we observed.

**Supplemental Figure 3. Topology of Mapper graphs generated from the subsampled data.** Samples from each node in the mapper graph are colored by plant family (A), stress (B), or tissue type (C ), using the subsampled data. The overall topology and sample distribution are similar to the Mapper graphs constructed with the full, unbalanced dataset, suggesting sample distribution is not a major factor in our analyses.

Together, this suggests that the backbone topology we observed is robust and not sensitive to biases in sample distribution or other related factors. This is a nice validation, and we have included this additional set of analyses in the results and added the new figures to the supplement.

2. Similarly, the authors restrict the analysis to 6,328 orthologous low copy genes that are conserved across the angiosperm species used. This is the only way to do the analysis, but it eliminates a lot of important genes from the analysis. For instance, most of the transcription factors and signaling pathway components controlling development are members of large protein families that are excluded from this analysis. This undoubtedly tilts the tissue results toward more core biological functions such as photosynthesis, ubiquitination, endomembrane trafficking, etc., instead of classic developmental regulators. The authors should discuss how this limitation of genes included in the analysis shapes and limits the outputs.

This is an excellent point, and we totally agree with the reviewer. Multigene families are where much of biological innovation comes from, but given their large size and rapid evolution, it is not easy to establish orthology between eudicots and monocots. In our revision, we discuss this limitation in the discussion as shown below:

“Although we observed a deeply conserved pattern of gene expression underlying plant form and function, our analyses capture a snapshot of the evolutionary innovations found in flowering plants. We used a set of low copy, conserved genes to enable comparisons of expression across species, and we had to exclude around ~70% of all plant genes. This includes most enzymes, transcription factors, and regulatory elements, which are mostly found in large, rapidly evolving or lineage specific gene families that cannot be resolved to high confidence orthologs across eudicots and monocots. Duplication and subsequent sub or neofunctionalization of these genes drives the evolution of new plant traits and developmental differences of plant organs. Single copy genes by contrast have deeply conserved functions in core metabolism, photosynthesis, and housekeeping processes that typically transcend tissue, species, and environmental changes. Given these limitations, it is somewhat surprising that our analyses were able to clearly separate tissue types and stresses despite missing information from most of the genes that should underlie these biological differences.”

3. This paper is introducing topological data analysis to most readers for the first time, so it is imperative to create a better schematic diagram explaining the analysis method to the reader. Figure 2a and 2b are not sufficient. The text lines ~167-172 describes a step wise process to arrive at the final shape. It would be worth creating a detailed step by step diagram illustrating the analysis process that directly relates to the steps described. Also please label the lens function with what it is, not just lens function #1.

This is an excellent point, and we hope the community considers adopting TDA more broadly when analyzing complex datasets such as gene expression, and central to this is making these methods more accessible. We have created a new schematic diagram that we hope explains the analysis methods more clearly. Below is the new schematic and we have added this to Figure 2:

In Figure 2, the lens functions have now been labeled for what they are as “lens function, y values” and “lens function, x values”.

Minor comments:

1. Line 54-55 "thousands of diverse plant species spanning over 900 million years of evolution" The sampled plants are all extant plants, so they do not span evolution. Maybe you mean some of the plants have diverged many million years ago?

Yes, we have corrected this to say ‘divergence’ instead of ‘evolution’.

2. What is in the "Other" category for tissue and stress?

These descriptions can be found in Supplemental Dataset 6, and this includes mostly tissues from mixed sources (e.g., whole seedling/plant) or tissues/stresses that were not clearly defined on the SRA metadata page or from the descriptions in the original papers.

3. Supplemental datasets 6 (mentioned in line 355) and 7 (mentioned in line 380) are missing from the submission (no link to them on the document).

We apologize for the omission, these two files can be found on the GitHub page, and we have included them in the revised submission.

4. For Stress the authors say there are 17 nodes in text, but there are 18 nodes labeled on the figure.

The reviewer is correct, there are 18 nodes in the figure, and we have corrected this in the text.

5. Please label which end is the stress end and which is the healthy end on Figure 4.

We have added labels of “Photosynthetic” and “Non-Photosynthetic” to the tissue Mapper graph colored by tissue, and we have added labels of “Healthy” and “Stressed” to the stress Mapper graph, colored by stress.

6. In Figure 2F, what does the box mean? Why isn't there an equivalent box on the tissue side, in Figure 2E?

The boxes indicate the Mapper graph constructions, resulting from the interval number and cover interval overlap parameters we chose, for further analysis. The missing box has now been added to the tissue Mapper graph and the figure legend has been updated.

7. Please check your color scheme for colorblind friendliness. Even for full color vision people, the pie charts in Figure 4 can be hard to see.

We were concerned about the ability to discern color for so many groups, especially for colorblind individuals, as well and consulted color brewer to optimize our color choice. For so many factor levels, distinguishing based on color will be difficult no matter our choice, and we believe we have optimized the palette to the extent that is possible based on simulating Protanopia and deuteranopia colorblindness in each figure in Illustrator.

8. Figure 3 seems to be mis-titled as stress lens, and is the tissue lens.

Thank you for catching this, we have fixed the error.

Rev. 2:

Palande et al report on a meta-analysis of transcriptomic data throughout the angiosperms. By simplifying highly dimensional gene expression data from many plant species with a topological simplification tool, Mapper, the authors have identified global features in the data reflecting developmental, taxonomic and environmental variables.

The approach is very original and the conceptual framework seems quite promising for synthesizing important features in the data. In principle, visualizations using dimensionality reduction can generate an ensemble of shapes, and as such interpreting these shapes could be misleading or even meaningless - I think this point should be emphasized in the introduction.

Thank you for the comment. We agree in principle that visualization techniques like the one used in this paper can generate a variety of structures, but that these structures alone are not necessarily meaningful without further investigation. We have clarified in the introduction that we are using these Mapper graphs of plant gene expression as a powerful and useful hypothesis generation tool that can reveal patterns that can be further analyzed within a more traditional hypothesis-testing framework. Our GO term enrichment results, for example, support the idea that the patterns identified in this analysis are meaningful.

How meaningful is the shape - what is the biological significance of having "loops, branch point, flares" (line 92)?

This is an excellent question. Below we have tried to explain the biological significance of shape, and we have included portions of this explanation in the introduction to help guide readers who might be unfamiliar with this type of mathematical approach.

Loops, branch points, and flares in TDA based Mapper graphs are visual representations of patterns, transitions, and outliers in the data. They provide insights into the topological structure and organization of the data, helping to identify clusters, subgroups, and potential anomalies. Loops in a Mapper graph are circular structures that represent recurring patterns or relationships in the data. They are formed when multiple data points are mapped to the same node in the graph, indicating that these points share similar topological properties. Branch points are locations in a Mapper graph where the graph splits into multiple paths. They occur when different subsets of data points exhibit distinct topological characteristics or when there are significant variations in the data. Branch points represent potential decision points or transitions in the data, where different clusters or groups emerge. Flares are extensions or protrusions that emerge from nodes in a Mapper graph. They occur when a subset of data points exhibits topological properties that are distinct from the majority of points in that node. Flares typically indicate outliers or subgroups within a larger cluster and can help identify regions of interest or anomalous behavior in the data.

I think the manuscript should emphasize more clearly what is the advantage of presenting the results with mapper graphs, and what novel conclusions could we draw (the source/sink continuum is apparent on the PC plot). It may be useful to look at other embedding algorithms (UMAP), and present different PCs/tSNE dimensions.

We have tried to emphasize this in the discussion section, and based on the feedback from the editor, we did not try other embedded algorithms, and we suspect they would yield similar results to PCA and t-SNE.

How biased is the dataset to species or stresses? It appears less biased to tissues, where most of the variation is detected. This is data with substantial sparsity and likely very sensitive to sampling.

This is an excellent point, and we have tried to address this in our response to reviewer 1. I have summarized the explanation below.

Assembling balanced datasets is currently a major challenge using public data, as most gene expression experiments are done in a few model or crop species with relatively narrow sampling across tissues or stresses. Balanced datasets simply do not exist for some of the factors we are exploring. To test if the unbalanced nature of our dataset is causing the patterns we observed, we downsampled the most frequent factor combinations and surveyed the effect it had on the Mapper graph topology. Our study has three factors: family, tissue, and stress with 16 families, 8 tissue types and 10 stresses. In total, 1280 unique 3-way combinations are possible (family+tissue+stress), but in our dataset, only 195 unique combinations are present and they have the following distribution. Based on this distribution, we chose a cutoff of 30 and downsampled the 30 most common factor combinations. This significantly reduced the sampling bias for family, tissue, and stress, but it did not eliminate them, as shown below. We then reran the Mapper algorithm using this downsampled dataset. The topology is quite similar, suggesting that biases in sample representation are not the major factor underlying the topological shape we observed.

We have added this downsampling analysis and associated figures to the results, and included a discussion of the sparsity of our data.

More generally, I am not sure if gene expression having any "structure" or "shape" is the most appropriate way to phrase the findings of the study. The resulting "shape" is a representation of the gene expression, rather that a guiding force for resulting phenotypes (line 95).

Here, we are referring to shape in a conceptual sense, and we have edited the introduction to better reflect that shape represents gene expression as described below:

“Surveys of gene expression capture tens of thousands of data points per sample, and this high dimensionality can be represented by a unique shape that underlies emergent biological features. This shape explains gene expression along evolutionary, developmental, and environmental trajectories, leading to innovations that have marked the successful adaptation and proliferation of plant species. To visualize this shape is to better understand what transcriptional profiles are possible, and to know the boundaries or constraints that permit or limit gene expression.”

Line 120 - Amborella is not a "basal" angiosperm - please rephrase to "sister of the rest of the angiosperms (for in-depth discussion, please check Stacey Smith's blog)

We thank the reviewer for catching this error, and have corrected the phrasing in text.

Rev. 3:

This study by Palande et al. entitled The topological shape of gene expression across the evolution of flowering plants harnesses the data dimensionality reduction procedure Mapper to derive a topological understanding of gene expression across diverse plant species. The study explores 2,671 SRA samples from 54 diverse plant species which are phylogenetically joined by 6,328 conserved orthogroups. When exploring the differences between tissue expression and stress expression (abiotic vs biotic) the authors interpret their projection and modelling outcomes as distinct and conserved expression patterns across angiosperms that delineate different tissue types or responses to biotic and abiotic stresses based on their agreement with a particular lens-function (e.g. length of residual vector).

While this study provides a promising opportunity to aggregate some of the vast sample space of the NCBI SRA database, it fails to deliver substantiating evidence and presents significant analytical, bioinformatic, and conceptual shortcomings that would need to be addressed in a major revision. If their conclusions still hold true after the sufficient scrutiny of (yet missing) negative controls and cross-sample data normalization, this study could provide value for a specialised audience that seeks to replicate their topological procedure for other SRA samples.

Major comments:

Overall, the two most important resources for reproducibility and study assessment were not provided for review by the authors: The final expression dataset (Suppl Data 7) and the GitHub repository (Data availability: The code, metadata, and raw datasets from this project are available on a dedicated GitHub page: <empty space>).

We apologize for the oversight here. Supplemental Dataset 7 is now available on GitHub and in the revision. This file is relatively large (~200 Mb), and we were not able to upload it in the first submission. The GitHub link was corrected, and is now active as well:

<https://github.com/PlantsAndPython/plant-evo-mapper>

Methodological shortcomings:

-	Assembly of gene expression data: While the intuition to generate TPM values based on a standardized pipeline is correct, the lack of any normalization across either tissues or across species or across stress conditions or across similar data quality is making the procedure of deriving comparable expression level estimates highly biased and insufficient. Without sufficient normalization and accounting for sequencing depth, coverage, sequencing technology conformity, contamination checks, batch effect correction, etc any observed topological pattern could be explained by these technical artifacts. Since none of these quality-control steps were performed and no controls to suggest otherwise were presented, I am not convinced that the presented topologies represent true biological signature. I have to assume that these patterns are largely driven by differences in their technical treatment and highly diverse experimental designs

This is an important point and we agree that standardization across the experiment is essential. We feel our analyses are highly standardized, statistically robust, and largely free of technical artifacts beyond the inherent noise of RNAseq data. Below we include a detailed summary of the various QC metrics we used and clarified this in text (including a new supplemental note about surrogate variable analysis).

We used Surrogate Variable Analysis (SVA) (Leek et al. 2012) to explore the effects of confounding technical variables on the publicly available SRA data assembled for this study. Briefly, we identified three primary variables of interest (tissue, stress, and family), which were fixed in the model used to estimate “surrogate variables” to minimize the amount of variability attributable to these primary variables captured by the estimated surrogate variables (see Supplementary Methods for Surrogate Variable Analysis). These surrogate variables represent unaccounted for technical variables impacting the dataset. Due to the breadth of families, stresses, and tissues analyzed, we do not have a full factorial design (i.e., there are combinations of family, stress, and tissue factor values for which there are no expression datasets). Because of this, SVA would remove variability due to our primary variables and their interactions. To get a sense of what kind of impact the surrogate variables might have on the dataset when removed, we estimated the correlation between the first order interactions between our primary variables and the surrogate variables identified by SVA. We identified 24 surrogate variables which individually captured between 53% and 98% of variation between BioProjects (Supplemental Figure 4). We also estimated the interaction terms between the tissue, family, and stress factor combinations that were present in the dataset and estimated how much of their variation was getting captured by the surrogate variables. Individual surrogate variables captured up to 14% of variation between stress conditions, up to 66% of variation between tissue conditions, and up to 63% of variation between families. For the interaction terms between primary variables, individual surrogate variables captured up to 83% of the variation between tissue and family combinations, up to 65% of the variation between stress and family combinations, and up to 71% of the variation between tissue and stress combinations. This suggests that even though stress, tissue, and family are treated as protected primary variables, there are underlying latent variables related to our primary variables and their interactions that may be important sources of biological variation being captured by the surrogate variables. Although individual surrogate variables could be selectively accounted for in downstream analyses in such a way that minimizes the removal of biological signal, this would be a highly subjective process. Moreover, due to our inability to precisely calculate the true correlation between our surrogate variables and interaction terms due to the fact that many factor combinations are missing, this would be statistically dubious as well.

Because the surrogate variables show substantial linear correlation with our primary variables and their interaction terms, the application of SVA would require eliminating substantial amounts of biological signal. Since the goal of our study is to identify heterogeneous patterns due to stress, tissue, and family within a high-dimensional gene expression dataset, SVA may not be appropriate for us to use. Alternatively, one could potentially minimize the loss of this signal by cherry-picking individual surrogate variables to include in downstream analysis, which would naturally introduce human bias. A third option would be to use an algorithm like ComBat-seq (Zhang, Parmigiani, and Johnson 2020) that relies on explicitly defined batches, which is problematic for the present study since the closest metadata for batch available for the studies gathered on SRA is the BioProject ID’s, but these are, at best, a proxy for batches of samples and are not sufficient to assess the technical variability or noise in the data. More broadly, as discussed in (Jaffe et al. 2015), such genomic data “cleaning” methods, by their very nature, delimit the observable features of the resulting datasets to those prespecified by the investigator. In our view, this limits their utility for broad exploratory analyses of the kind described in this study. For all the above reasons, we opted to not use SVA, ComBat, or related techniques prior to downstream analyses. These shortcomings also emphasize the need for tools like Mapper that can, as shown in this manuscript, reveal patterns that are amenable to downstream analysis.

The raw TPM values have some degree of standardization by library size (i.e., number of reads per sample), and we transformed all expression values by Z-score prior to any downstream analyses. The Z-score enables cross-species comparisons as values within each dataset are normalized to a common scale based on their standard deviation and mean rather than absolute values. Dimensionality reduction (t-SNE and PCA) from the z-score transformed expression in Figure 1 shows a clear separation of different plant tissues across species, suggesting we are identifying real developmental patterns in our dataset and not technological artifacts. Labeling the samples by technology, year the dataset was published, BioProject, or other variables showed no correlation. Tissue patterns are quite clear, and there is no reasonable way that technical artifacts could be causing this delineation as any artifacts or variability would be found within all sample types and species and not within a specific factor such as tissue, stress, or species that could create a misleading pattern. Furthermore, GO term analysis identified sets of genes that are consistent with the lens function we were using, such as photosynthetic genes delineating leaves from other tissues and stress responsive genes delineating healthy from unhealthy tissues. If our analyses were picking up on technical artifacts instead of biological patterns, there should be no enriched GO terms that are consistent with our classification. We hope this clarifies the concerns raised by this reviewer and showcases the utility of our dataset and approach.

-	Mathematical basis of topological data analysis: The authors present the Mapper approach as the only alternative compared to established dimensionality reduction methods that was able to present them with patterns they felt comfortable to interpret (p. 8 "We first tested traditional dimensionality reduction and clustering-based approaches but found they were largely ineffective and unable to clearly resolve samples. Instead, we used a novel topological framework to compare samples and test for evolutionary conservation."). How can the authors ensure that traditional dimensionality reduction and clustering-based approaches didn't fail, because they (in fact intrinsically) captured the bias of the non-standardized data? How would the authors argue against the argument that they cherry-picked a method that allowed them to show any pattern? The Mapper approach is also known to have analogous weaknesses as traditional dimensionality reduction methods such as determining an

appropriate and agnostic number of overlapping intervals used to cover the data and the robustness of diverse clustering algorithm (and distance metric) able to consistently group similar points together. I interpret the claim "We experimented with a range of value lengths of the intervals and the size of the overlap to identify the values that produced relatively stable mapper graphs." as visually/manually cherry-picking the most favourable topologies rather than relying on a robust and objective metric.

We are receptive to the concern that, due to the lack of concordance between dimensionality approaches like PCA and our Mapper graphs and the lack of a robust hyperparameter tuning procedure for TDA, it may appear that we are cherry-picking favorable topologies to present. We believe it is reasonable to assume that if the structures we are presenting are either (a) highly sensitive to the exact set of samples in the dataset, or (b) not reproducible using intuitive related, but distinct, lens functions, this would undermine the strength of our results. However, as shown in our responses demonstrating robustness to downsampling and the supplemental data showing results from using roots as a lens, our results are robust in both cases. This gives us confidence in the results we are presenting.

We tested the most commonly used dimensionality reduction approaches for expression-based datasets including MDS, t-sne, and PCA as well as hierarchical clustering approaches, and it is certainly possible that other methodologies could produce similar topologies to our TDA based analyses, but we are quite skeptical. We agree with the reviewer that these analyses may be picking up technical differences between experiments such as variations in how, when, and which exact tissues were collected, differences in genotype, or variation in the duration or magnitude of stress. This is somewhat supported by our results from the surrogate variable analysis, which was unable to remove these artifacts without removing the variables we were testing (e.g, tissues, species, or stresses). This is really the core finding of our manuscript: TDA can find hidden biological structure in complex, noisy datasets that is missed by traditional dimensionality reduction.

The fact that furthermore "The clustering was performed using DBSCAN, a commonly used clustering algorithm in Mapper [14]." without demonstrating the robustness of topologies to differences in clustering methods further strengthens my suspicion. The claim that other studies also used the Mapper approach on gene expression data despite the open problem in TDA research to optimize Mapper parameters is not convincing at this stage where traditional dimensionality reduction methods fail.

While DBSCAN is a commonly used clustering algorithm in Mapper, we acknowledge that demonstrating the robustness of topologies to different clustering methods would be valuable. In our study, we used DBSCAN as it has been shown to perform well in various applications and has been widely adopted in the field. DBSCAN doesn't require specifying the number of clusters, so that eliminates testing most other clustering algorithms.

Regarding the claim that other studies have also used the Mapper approach despite the open problem in TDA research to optimize Mapper parameters, we understand your skepticism. However, it is worth noting that the Mapper approach has proven to be effective in capturing topological structures in diverse datasets, including gene expression data. While traditional dimensionality reduction methods may struggle to handle the complexity and non-linearity of gene expression data, Mapper provides a valuable alternative for uncovering meaningful patterns. We did provide some parameter optimization across cover intervals and interval number for both of the lens functions, and these had little to no effect on the backbone Mapper graph, so again, we feel our results are robust.

-	For the claim that most of the community assumes that biotic and abiotic stress are evolutionarily independent (not clear to me what this means), no reference is presented and their finding that biotic and abiotic stress samples (therefore unexpectedly) showed similar topologies/patterns/distributions further illustrates the importance to standardize and normalize across SRA samples, tissues, species etc. The fact that biotic and abiotic stress samples show similar patterns/topologies may simply be the result of the fact that most stress responses involve a much smaller number of genes than tissue development or housekeeping and thus technical artifacts are more pronounced under these (tissue, housekeeping, non-stress) conditions. Since no control experiments are provided, I am not convinced that these stress similarities are in fact true biological signatures.

This is an excellent point. We agree with the reviewer, the claim that biotic and abiotic stress are evolutionarily independent is contentious and unclear, and we have revised this in the text for clarity and to provide support for our statements. We have modified this section as shown below:

“Abiotic and biotic stress responses have been mostly studied in isolation, but they typically co-occur in natural environments, and they have overlapping signaling, hormonal, and network responses in plants (reviewed in (Rejeb et al. 2014)). The topological shape of gene expression points to a shared set of pathways or perturbations that define if a tissue is healthy or stressed. Environmental stresses broadly disrupt photosynthesis, core metabolic and cellular functions as either a direct response to physical trauma, or in preparation for defense or resilience. These changes may serve as the backbone of the topological shape we observed for the stress lens.”

Most stress responses involve massive transcriptional reprogramming, especially when the stress is severe, and typically thousands of genes have differential expression or regulation under stress compared to control. This includes many genes with roles in steady state processes such as photosynthesis, metabolism, growth, and core cellular characteristics, as well as stress signaling and numerous downstream stress response pathways. Together, this intense shift likely underlies the topological shape we observed. Furthermore, we observed stressed samples from multiple tissues clustering together within the mapper graph and some separation of individual stresses such as high light or cold, suggesting that these signals are far stronger than any technical or background effects.

We disagree that control experiments are not provided as nearly every stress sample has a comparable healthy or control sample and these are used as the foundation for the stress lens function.
