# Supplementary material for: Topological data analysis reveals a core gene expression backbone that defines form and function across flowering plants
Source: PLoS Biol. 2023 Dec 5;21(12):e3002397. doi: 10.1371/journal.pbio.3002397 (PMC10723737; doi:10.1371/journal.pbio.3002397)
Supplement: Attachment — Submitted filename: PLoS Biology reviewer responses 8-30-23.docx [file pbio.3002397.s010.docx]

Reviewers' comments

Rev. 3:

I appreciate the authors efforts to address my concerns. Some of my concerns were successfully approached and are now resolved, but a few major concerns remain unresolved which need further attention. I therefore recommend to address these concerns in full in a minor revision.

Below we have tried to address the remaining comments from Reviewer 3 in blue.

Rev. 3 (previous comments):

This study by Palande et al. entitled The topological shape of gene expression across the evolution of flowering plants harnesses the data dimensionality reduction procedure Mapper to derive a topological understanding of gene expression across diverse plant species. The study explores 2,671 SRA samples from 54 diverse plant species which are phylogenetically joined by 6,328 conserved orthogroups. When exploring the differences between tissue expression and stress expression (abiotic vs biotic) the authors interpret their projection and modelling outcomes as distinct and conserved expression patterns across angiosperms that delineate different tissue types or responses to biotic and abiotic stresses based on their agreement with a particular lens-function (e.g. length of residual vector).

While this study provides a promising opportunity to aggregate some of the vast sample space of the NCBI SRA database, it fails to deliver substantiating evidence and presents significant analytical, bioinformatic, and conceptual shortcomings that would need to be addressed in a major revision. If their conclusions still hold true after the sufficient scrutiny of (yet missing) negative controls and cross-sample data normalization, this study could provide value for a specialised audience that seeks to replicate their topological procedure for other SRA samples.

Major comments:

Overall, the two most important resources for reproducibility and study assessment were not provided for review by the authors: The final expression dataset (Suppl Data 7) and the GitHub repository (Data availability: The code, metadata, and raw datasets from this project are available on a dedicated GitHub page: <empty space>).

Authors: We apologize for the oversight here. Supplemental Dataset 7 is now available on GitHub and in the revision. This file is relatively large (~200 Mb), and we were not able to upload it in the first submission. The GitHub link was corrected, and is now active as well: <https://github.com/PlantsAndPython/plant-evo-mapper>

Methodological shortcomings:

Authors: Assembly of gene expression data: While the intuition to generate TPM values based on a standardized pipeline is correct, the lack of any normalization across either tissues or across species or across stress conditions or across similar data quality is making the procedure of deriving comparable expression level estimates highly biased and insufficient. Without sufficient normalization and accounting for sequencing depth, coverage, sequencing technology conformity, contamination checks, batch effect correction, etc any observed topological pattern could be explained by these technical artifacts. Since none of these quality-control steps were performed and no controls to suggest otherwise were presented, I am not convinced that the presented topologies represent true biological signature. I have to assume that these patterns are largely driven by differences in their technical treatment and highly diverse experimental designs

This is an important point and we agree that standardization across the experiment is essential. We feel our analyses are highly standardized, statistically robust, and largely free of technical artifacts beyond the inherent noise of RNAseq data.

Rev-3: While I appreciate the authors' confidence in the SRA database, would it be possible to specifically state how normalization ACROSS diverse SRA samples were performed to provide concrete evidence for their strong claim that "[…] our analyses are highly standardized, statistically robust, and largely free of technical artifacts beyond the inherent noise of RNAseq data"? Did the authors rely on the "SRA Normalized Format" (default, see e.g. <https://www.ncbi.nlm.nih.gov/sra/docs/data-format-faq/>) as input to their analyses? If yes, did the authors check whether the samples giving strongest biological signature (especially the ones not captured by alternative dimensionality reduction approaches) still have a high "original quality score" in the non-SRA normalized samples (see FAQs in the previous link)? This important analysis detail should be made clear in the manuscript.

Authors: Below we include a detailed summary of the various QC metrics we used and clarified this in text (including a new supplemental note about surrogate variable analysis).

We used Surrogate Variable Analysis (SVA) (Leek et al. 2012) to explore the effects of confounding technical variables on the publicly available SRA data assembled for this study. Briefly, we identified three primary variables of interest (tissue, stress, and family), which were fixed in the model used to estimate "surrogate variables" to minimize the amount of variability attributable to these primary variables captured by the estimated surrogate variables (see Supplementary Methods for Surrogate Variable Analysis). These surrogate variables represent unaccounted for technical variables impacting the dataset. Due to the breadth of families, stresses, and tissues analyzed, we do not have a full factorial design (i.e., there are combinations of family, stress, and tissue factor values for which there are no expression datasets). Because of this, SVA would remove variability due to our primary variables and their interactions. To get a sense of what kind of impact the surrogate variables might have on the dataset when removed, we estimated the correlation between the first order interactions between our primary variables and the surrogate variables identified by SVA. We identified 24 surrogate variables which individually captured between 53% and 98% of variation between BioProjects (Supplemental Figure 4). We also estimated the interaction terms between the tissue, family, and stress factor combinations that were present in the dataset and estimated how much of their variation was getting captured by the surrogate variables. Individual surrogate variables captured up to 14% of variation between stress conditions, up to 66% of variation between tissue conditions, and up to 63% of variation between families. For the interaction terms between primary variables, individual surrogate variables captured up to 83% of the variation between tissue and family combinations, up to 65% of the variation between stress and family combinations, and up to 71% of the variation between tissue and stress combinations. This suggests that even though stress, tissue, and family are treated as protected primary variables, there are underlying latent variables related to our primary variables and their interactions that may be important sources of biological variation being captured by the surrogate variables. Although individual surrogate variables could be selectively accounted for in downstream analyses in such a way that minimizes the removal of biological signal, this would be a highly subjective process. Moreover, due to our inability to precisely calculate the true correlation between our surrogate variables and interaction terms due to the fact that many factor combinations are missing, this would be statistically dubious as well.

Because the surrogate variables show substantial linear correlation with our primary variables and their interaction terms, the application of SVA would require eliminating substantial amounts of biological signal. Since the goal of our study is to identify heterogeneous patterns due to stress, tissue, and family within a high-dimensional gene expression dataset, SVA may not be appropriate for us to use. Alternatively, one could potentially minimize the loss of this signal by cherry-picking individual surrogate variables to include in downstream analysis, which would naturally introduce human bias. A third option would be to use an algorithm like ComBat-seq (Zhang, Parmigiani, and Johnson 2020) that relies on explicitly defined batches, which is problematic for the present study since the closest metadata for batch available for the studies gathered on SRA is the BioProject ID's, but these are, at best, a proxy for batches of samples and are not sufficient to assess the

technical variability or noise in the data. More broadly, as discussed in (Jaffe et al. 2015), such genomic data "cleaning" methods, by their very nature, delimit the observable features of the resulting datasets to those prespecified by the investigator. In our view, this limits their utility for broad exploratory analyses of the kind described in this study. For all the above reasons, we opted to not use SVA, ComBat, or related techniques prior to downstream analyses. These shortcomings also emphasize the need for tools like Mapper that can, as shown in this manuscript, reveal patterns that are amenable to downstream analysis.

Rev-3: Thank you for this detailed assessment of why their input samples are too heterogeneous or too sparsely sampled for sufficient batch effect correction. This was actually one of my major concerns of this study that if classic batch-effect smoothing/removal methods fail, dimensionality reduction will only project this shortcoming into a lower dimensional space. The authors' argument that since their input data represents an insufficient sampling to enable various batch effect removal methods, their dimensionality reduction approach can reveal true biological patterns is not clear to me, since no data or evidence is presented for this claim. The core principle of batch effect removal relies on exploring the nature of the variance in sufficiently sampled data and so does dimensionality reduction. Thus, both methods should be sensitive to insufficient sampling and data heterogeneity. Do the authors disagree?

Response: We agree with the reviewer that dimensionality reduction, similar to SVA, is sensitive to insufficient sampling and heterogeneity. As we discuss in our previous response, the sparsity and heterogeneity of the dataset make it so application of SVA and related methods would necessarily remove substantial amounts of signal related to our primary variables of interest (tissue and stress). Given that the aim of this study is to examine, through an exploratory data analysis lens, well-conserved gene expression patterns related to tissue-type and stress conditions across a diverse collection of samples, we therefore cannot apply such techniques. What we demonstrate in this study, and elaborate on in our responses to the reviewer’s other comments, is the extraction of meaningful biological signals from a complex and sparse dataset using TDA despite these shortcomings of the dataset. To summarize, although both dimensionality reduction and more traditional batch-effect correction are affected by data sparsity and heterogeneity, the former would have precluded us from performing any meaningful analyses due to an unavoidable loss of signal in the dataset, whereas the latter allowed us to observe patterns which we then corroborated with further confirmatory analyses. For support of this latter claim, please refer to the discussion of the robustness of our findings to down-sampling, parameter tuning, etc.

Authors: The raw TPM values have some degree of standardization by library size (i.e., number of reads per sample), and we transformed all expression values by Z-score prior to any downstream analyses.

Rev-3: Thank you for clarifying. Was this Z-score standardization performed on the "SRA Normalized Format" (default, see e.g. <https://www.ncbi.nlm.nih.gov/sra/docs/data-format-faq/>) or on the SRA raw samples (non-SRA normalized samples)? This important analysis detail should be made clear in the manuscript.

Response: Z-score normalization was performed on the raw TPMs from the SRA data, and not normalized NCBI format. We have clarified this in text.

Authors: The Z-score enables cross-species comparisons as values within each dataset are normalized to a common scale based on their standard deviation and mean rather than absolute values. Dimensionality reduction (t-SNE and PCA) from the z-score transformed expression in Figure 1 shows a clear separation of different plant tissues across species, suggesting we are identifying real developmental patterns in our dataset and not technological artifacts. Labeling the samples by technology, year the dataset was published, BioProject, or other variables showed no correlation.

Rev-3: Excellent! Would it be possible to add this analysis ("Labeling the samples by technology, year the dataset was published, BioProject, or other variables showed no correlation.") as Supplementary Figures? This should give readers more confidence in the methodology.

Response: We agree, this is an important consideration. These plots can be regenerated using any of these factors, and we have provided the raw data (containing BioProjects) and our python code to rerun this analysis in reproducible, easy to use Jupyter notebooks on our dedicated GitHub page: <https://github.com/PlantsAndPython/plant-evo-mapper> which we have linked in the paper.

Authors: Tissue patterns are quite clear, and there is no reasonable way that technical artifacts could be causing this delineation as any artifacts or variability would be found within all sample types and species and not within a specific factor such as tissue, stress, or species that could create a misleading pattern. Furthermore, GO term analysis identified sets of genes that are consistent with the lens function we were using, such as photosynthetic genes delineating leaves from other tissues and stress responsive genes delineating healthy from unhealthy tissues. If our analyses were picking up on technical artifacts instead of biological patterns, there should be no enriched GO terms that are consistent with our classification.

Rev-3: Thank you for performing this quality control analysis. I agree with the authors that this is promising evidence. Did the authors have a chance to confirm this analysis with a negative control whereby samples with clear signatures of technical artifacts do not pick up enriched GO-terms that would be consistent with their lens function? If this is indeed the case, then readers will appreciate this analysis by placing more confidence in their method.

Response: Thank you for your thoughtful suggestion regarding the inclusion of a negative control to further validate our GO-term enrichment findings. While we recognize the value such a control could provide, unfortunately, it is not easily feasible within the context of our current dataset. Given the inherent data sparsity and the multifactorial nature of our study, identifying a set of samples that carry clear signatures of technical artifacts for a negative control is particularly challenging. Therefore, we have not been able to perform this specific analysis for the manuscript.

Authors: We hope this clarifies the concerns raised by this reviewer and showcases the utility of our dataset and approach.

- Mathematical basis of topological data analysis: The authors present the Mapper approach as the only alternative compared to established dimensionality reduction methods that was able to present them with patterns they felt comfortable to interpret (p. 8 "We first tested traditional dimensionality reduction and clustering-based approaches but found they were largely ineffective and unable to clearly resolve samples. Instead, we used a novel topological framework to compare samples and test for evolutionary conservation."). How can the authors ensure that traditional dimensionality reduction and clustering-based approaches didn't fail, because they (in fact intrinsically) captured the bias of the non-standardized data? How would the authors argue against the argument that they cherry-picked a method that allowed them to show any pattern? The Mapper approach is also known to have analogous weaknesses as traditional dimensionality reduction methods such as determining an

appropriate and agnostic number of overlapping intervals used to cover the data and the robustness of diverse clustering algorithm (and distance metric) able to consistently group similar points together. I interpret the claim "We experimented with a range of value lengths of the intervals and the size of the overlap to identify the values that produced relatively stable mapper graphs." as visually/manually cherry-picking the most favourable topologies rather than relying on a robust and objective metric.

Authors: We are receptive to the concern that, due to the lack of concordance between dimensionality approaches like PCA and our Mapper graphs and the lack of a robust hyperparameter tuning procedure for TDA, it may appear that we are cherry-picking favorable topologies to present.

Rev-3: Thank you for confirming that TDA lacks a robust hyperparameter tuning procedure. My major concern in this context was not that the authors cherry-picked their results, but that users of their method when broadly employed to various datasets may feel inclined to cherry-pick in the absence of an unbiased hyperparameter tuning procedure. This point needs to be extensively discussed in the main manuscript and clear guidelines presented to avoid (un)intentional cherry-picking by the end user.

Response: This is an excellent point, and your concerns about the absence of a robust hyperparameter tuning procedure in TDA are well-founded and touch upon a significant issue in machine learning and data science more broadly. The risk of cherry-picking results, intentionally or unintentionally, is elevated when there are no clear guidelines for hyperparameter selection. It could compromise the validity and generalizability of the findings, potentially leading to misleading conclusions. We have added a paragraph to the discussion to include these important caveats.

Authors: We believe it is reasonable to assume that if the structures we are presenting are either (a) highly sensitive to the exact set of samples in the dataset, or (b) not reproducible using intuitive related, but distinct, lens functions, this would undermine the strength of our results. However, as shown in our responses demonstrating robustness to downsampling and the supplemental data showing results from using roots as a lens, our results are robust in both cases. This gives us confidence in the results we are presenting.

Rev-3: While I agree with this statement and appreciate the analysis, the concern lies rather with the fact that less nuanced input data (as could be explored by users of the TDA method) could demonstrate less robustness (which was never tested by the end user). Thus, this type of "robustness confirmation analysis" should be clearly communicated to the user.

Response: This is an important point, and we have tried to clarify this caveat in the discussion.

Authors: We tested the most commonly used dimensionality reduction approaches for expression-based datasets including MDS, t-sne, and PCA as well as hierarchical clustering approaches, and it is certainly possible that other methodologies could produce similar topologies to our TDA based analyses, but we are quite skeptical. We agree with the reviewer that these analyses may be picking up technical differences between experiments such as variations in how, when, and which exact tissues were collected, differences in genotype, or variation in the duration or magnitude of stress. This is somewhat supported by our results from the surrogate variable analysis, which was unable to remove these artifacts without removing the variables we were testing (e.g, tissues, species, or stresses).

Rev-3: I greatly appreciate that the authors confirm my major concerns and that established dimensionality reduction methods are not robust to technical or sampling artifacts.

Authors: This is really the core finding of our manuscript: TDA can find hidden biological structure in complex, noisy datasets that is missed by traditional dimensionality reduction.

Rev-3: It remains unclear to me what the actual evidence is that TDA can find hidden biological structure not captured by traditional methods. The only evidence presented is the GO-term analysis that matches the lens function. But for this analysis no control is presented. It is therefore paramount to present this control (see corresponding point above) to have at least some form of evidence for this claim.

Response: The inherent topology of the Mapper graph demonstrates its effectiveness in uncovering previously undetected biological structures. Specifically, the nodes in the graph exhibit a distinct gradient ranging from photosynthetic to non-photosynthetic tissues, as well as from stressed to non-stressed tissues across multiple species. Such patterns were not discernible using conventional dimensionality reduction techniques. If the Mapper graph was not capturing meaningful biological structure, we would anticipate the nodes to reflect a random assortment of variables, such as tissue types or stress levels, rather than the coherent patterns that we have observed. While Gene Ontology (GO) term analyses have their limitations, they nonetheless reveal significant enrichment patterns. These patterns further substantiate the functionally relevant separation of factors indicated by the Mapper graph.

Authors: The fact that furthermore "The clustering was performed using DBSCAN, a commonly used clustering algorithm in Mapper [14]." without demonstrating the robustness of topologies to

differences in clustering methods further strengthens my suspicion. The claim that other studies also used the Mapper approach on gene expression data despite the open problem in TDA research to optimize Mapper parameters is not convincing at this stage where traditional dimensionality reduction methods fail.

While DBSCAN is a commonly used clustering algorithm in Mapper, we acknowledge that demonstrating the robustness of topologies to different clustering methods would be valuable. In our study, we used DBSCAN as it has been shown to perform well in various applications and has been widely adopted in the field. DBSCAN doesn't require specifying the number of clusters, so that eliminates testing most other clustering algorithms.

Regarding the claim that other studies have also used the Mapper approach despite the open problem in TDA research to optimize Mapper parameters, we understand your skepticism. However, it is worth noting that the Mapper approach has proven to be effective in capturing topological structures in diverse datasets, including gene expression data. While traditional dimensionality reduction methods may struggle to handle the complexity and non-linearity of gene expression data, Mapper provides a valuable alternative for uncovering meaningful patterns. We did provide some parameter optimization across cover intervals and interval number for both of the lens functions, and these had little to no effect on the backbone Mapper graph, so again, we feel our results are robust.

- For the claim that most of the community assumes that biotic and abiotic stress are evolutionarily independent (not clear to me what this means), no reference is presented and their finding that biotic and abiotic stress samples (therefore unexpectedly) showed similar topologies/patterns/distributions further illustrates the importance to standardize and normalize across SRA samples, tissues, species etc. The fact that biotic and abiotic stress samples show similar patterns/topologies may simply be the result of the fact that most stress responses involve a much smaller number of genes than tissue development or housekeeping and thus technical artifacts are more pronounced under these (tissue, housekeeping, non-stress) conditions. Since no control experiments are provided, I am not convinced that these stress similarities are in fact true biological signatures.

This is an excellent point. We agree with the reviewer, the claim that biotic and abiotic stress are evolutionarily independent is contentious and unclear, and we have revised this in the text for clarity and to provide support for our statements. We have modified this section as shown below:

"Abiotic and biotic stress responses have been mostly studied in isolation, but they typically co-occur in natural environments, and they have overlapping signaling, hormonal, and network responses in plants (reviewed in (Rejeb et al. 2014)). The topological shape of gene expression points to a shared set of pathways or perturbations that define if a tissue is healthy or stressed. Environmental stresses broadly disrupt photosynthesis, core metabolic and cellular functions as either a direct response to physical trauma, or in preparation for defense or resilience. These changes may serve as the backbone of the topological shape we observed for the stress lens."

Most stress responses involve massive transcriptional reprogramming, especially when the stress is severe, and typically thousands of genes have differential expression or regulation under stress compared to control. This includes many genes with roles in steady state processes such as photosynthesis, metabolism, growth, and core cellular characteristics, as well as stress signaling and numerous downstream stress response pathways. Together, this intense shift likely underlies the topological shape we observed. Furthermore, we observed stressed samples from multiple tissues clustering together within the mapper graph and some separation of individual stresses such as high light or cold, suggesting that these signals are far stronger than any technical or background effects.

We disagree that control experiments are not provided as nearly every stress sample has a comparable healthy or control sample and these are used as the foundation for the stress lens function.
